# Ultrafast Dynamics of Sb-Corroles: A Combined Vis-Pump Supercontinuum Probe and Broadband Fluorescence Up-Conversion Study

**DOI:** 10.3390/molecules22071174

**Published:** 2017-07-13

**Authors:** Clark Zahn, Till Stensitzki, Mario Gerecke, Alexander Berg, Atif Mahammed, Zeev Gross, Karsten Heyne

**Affiliations:** 1 Department of Physics, Free University Berlin, Arnimallee 14, D-14195 Berlin, Germany; clark.zahn@fu-berlin.de (C.Z.); tillsten@zedat.fu-berlin.de (T.S.); 2 Department of Chemistry, Humboldt-Universität zu Berlin, Brook-Taylor-Str. 2, D-12489 Berlin, Germany; Mario.gerecke@chemie.hu-berlin.de; 3 Institute of Chemistry, The Hebrew University of Jerusalem, Jerusalem 91904, Israel; alexander.berg@mail.huji.ac.il; 4 Schulich Faculty of Chemistry, Technion-Israel Institute of Technology, Haifa 3200008, Israel; chatif@technion.ac.il (A.M.); chr10zg@technion.ac.il (Z.G.)

**Keywords:** photoreaction, triplet formation, femtosecond spectroscopy, fluorescence up-conversion, corrole dynamics, tetrapyrrole, ultrafast relaxation, intersystem crossing

## Abstract

Corroles are a developing class of tetrapyrrole-based molecules with significant chemical potential and relatively unexplored photophysical properties. We combined femtosecond broadband fluorescence up-conversion and fs broadband Vis-pump Vis-probe spectroscopy to comprehensively characterize the photoreaction of 5,10,15-tris-pentafluorophenyl-corrolato-antimony(V)-*trans*-difluoride (Sb-tpfc-F_2_). Upon fs Soret band excitation at ~400 nm, the energy relaxed almost completely to Q band electronic excited states with a time constant of 500 ± 100 fs; this is evident from the decay of Soret band fluorescence at around 430 nm and the rise time of Q band fluorescence, as well as from Q band stimulated emission signals at 600 and 650 nm with the same time constant. Relaxation processes on a time scale of 10 and 20 ps were observed in the fluorescence and absorption signals. Triplet formation showed a time constant of 400 ps, with an intersystem crossing yield from the Q band to the triplet manifold of between 95% and 99%. This efficient triplet formation is due to the spin-orbit coupling of the antimony ion.

## 1. Introduction

Corroles are a developing class of tetrapyrrole-based photosensitizers with relatively unexplored photophysical properties [1,2,3,4]. The simple and efficient procedure of corrole synthesis, combined with the pre-tuning of physical and chemical characteristics by varying the peripheral substituents [5,6,7,8], central metal [6,9,10], and axial ligands [11,12], has revived substantial interest in employing these contracted porphyrinoids in various fields. A wide range of applications has been reported: corroles are used in dye-sensitized solar cells [9,13], as photosensitizers in photodynamic therapy and photodynamic detection [14,15,16,17], for the photodynamic inactivation of mold fungi and green algae [18,19], for regular and sophisticated optical imaging [20,21], for the formation of singlet oxygen for catalysis [22,23,24] and for corrole-based electron and energy transfer systems [25,26,27]. Since many of the processes involving corroles proceed via transient paramagnetic states, revealing the mechanisms and parameters of these states as well as identifying their reaction pathways is central to the optimized application of corroles [8,28,29,30,31,32,33,34,35,36,37,38,39]. However, there are only a few in-depth reports tracking the photoreaction of corroles [35,39,40,41,42,43,44,45,46]. A combined study on the ultrafast electronic and vibrational dynamics of a brominated corrole system demonstrated intersystem crossing to the triplet manifold with a time constant of 100 ps and a quantum yield of close to one [39]. Whether efficient intersystem crossing can only be achieved by heavy atom insertion at the macrocycle ring or can also be realized by varying the peripheral substituents and/or axial ligands, causing macrocycle distortion and thus leading to changes in the electronic structure of the complex, is not fully understood. Moreover, it is also unclear if the absorption spectra of the triplet and singlet excited states are similar for most corroles or if the spectra are sensitive to metal ions and ligands. It has been reported that excitation of the Soret band at around 400 nm leads to energy relaxation into Q band states between 500 nm and 700 nm within a picosecond, accompanied by the rise of Q band fluorescence [37]. Whether an intersystem crossing takes place during Soret band to Q band transitions is unknown.

**Figure 1:**
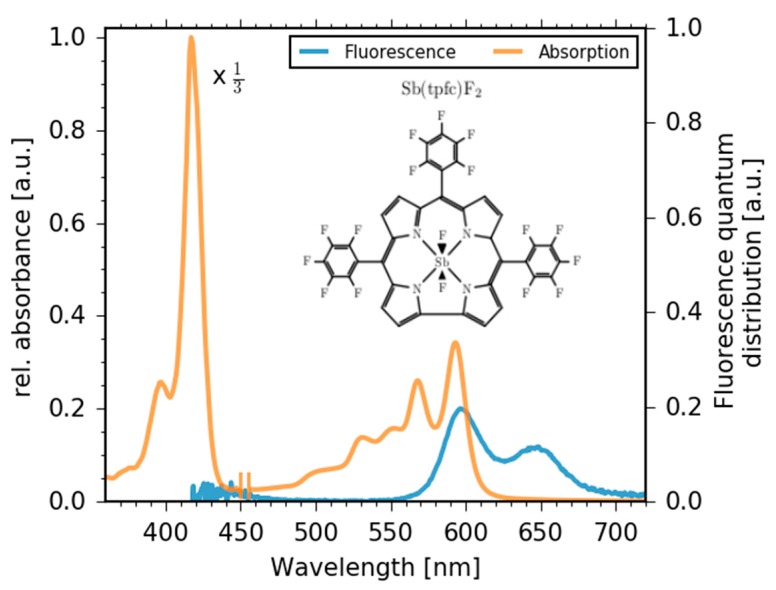
Steady-state absorption (orange line) and stationary fluorescence (blue line) of 5,10,15-tris-pentafluorophenyl-corrolato-antimony(V)-*trans*-difluoride (Sb-tpfc-F_2_). Inset: Schematic of Sb-tpfc-F_2_. Note, the short wavelength part of the absorption spectrum up to 450 nm is scaled-down by a factor of 3.

Here we report a study into the ultrafast electronic dynamics of a 5,10,15-tris-pentafluorophenyl-corrolato trianion (tpfc) chelating an antimony metal ion. The antimony corrole is further stabilized by two axial fluorides forming a Sb-tpfc-F_2_ complex (see inset of Figure 1). The ultrafast dynamics were investigated by a combination of two complementary time-resolved spectroscopy methods: broadband fluorescence up-conversion and broadband absorption spectroscopy.

## 2. Results

Steady-state absorption (orange line) and stationary fluorescence spectra (blue line) are displayed in Figure 1. The Soret band peaks at 410 nm, while two Q band peaks are clearly visible at 565 nm and 590 nm. The fluorescence spectrum (blue line) exhibits two prominent peaks at 596 nm and 650 nm. Moreover, a weak fluorescence contribution is indicated around 450 nm.

### 2.1. Transient Fluorescence Measurements

**Figure 2:**
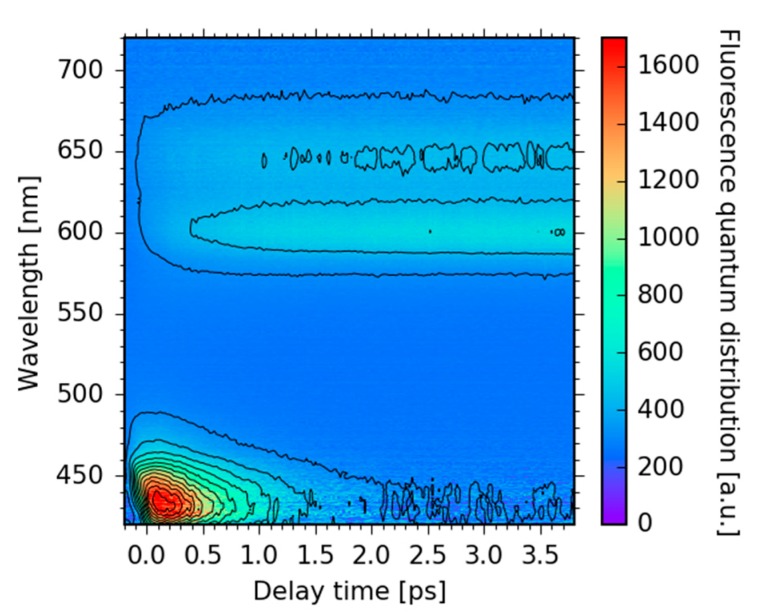
Fluorescence map of Sb-tpfc-F_2_ upon excitation at 400 nm. The fluorescence wavelength in nm is plotted as a function of delay time in ps. Fluorescence quantum distributions are color coded.

Dynamics of electronic transitions show absorption and emission or fluorescence signals. A clean detection of fluorescence signals is only possible by way of time-resolved fluorescence measurements. Here, we applied Vis-pump/broadband up-conversion fluorescence spectroscopy with a system response of 100 fs to track fluorescence dynamics in a spectral range from 417 to 720 nm (14,000 to 24,000 cm^−1^) over time. In Figure 2, the time-resolved fluorescence map is plotted for delay times up to 4 ps after Soret band excitation at 400 nm (25,000 cm^−1^).

**Figure 3:**
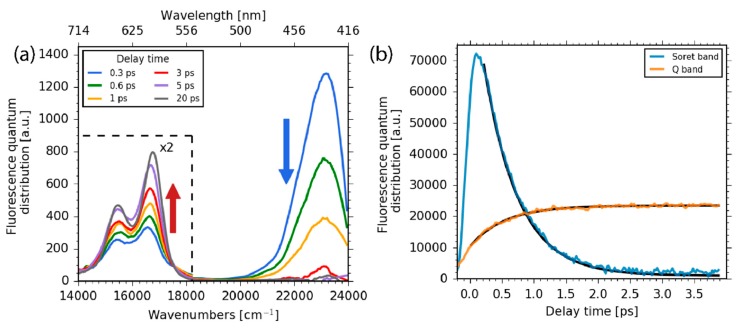
(**a**) Fluorescence spectra for selected delay times. The decay of the Soret band (blue arrow) and the subsequent rise of Q band fluorescence (red arrow) is clearly visible. For delay times of 5–20 ps, the two Q band fluorescence peaks shift to higher frequencies; (**b**) The integrated fluorescence transient of the Soret band region from 417 to 500 nm (blue line) and mono-exponential simulation with a time constant of 0.5 ± 0.1 ps; integrated fluorescence transients of the Q band region (orange line) with a mono-exponential rise time constant of 0.5 ± 0.1 ps.

For early delay times, of up to 500 fs, significant fluorescence is detected only at around 430 nm (23,000 cm^−1^), indicating direct fluorescence from the Soret band electronic states, violating Kasha’s rule. The transient of the Soret band fluorescence is plotted in Figure 3b and shows a mono-exponential decay with a time constant of 0.5 ± 0.1 ps. As depicted in Figure 2, the decay of the Soret band fluorescence is accompanied by the rise of the fluorescence in the Q band at around 600 nm (16,600 cm^−1^). Closer inspection of the Q band fluorescence shows a rise of the fluorescence in the spectral range from 570 to 700 nm (15,000 to 17,500 cm^−1^, see Figure 3a) with a time constant of 0.5 ± 0.1 ps (see Figure 3b). A similar decay of the Soret band fluorescence accompanied by a rise of the Q band fluorescence on a sub-picosecond time scale was previously observed in the case of aluminum corroles with two pyridine ligands (Al-tpfc-py_2_) in a study of selected fluorescence wavelengths [37]. Here, we can also track subtle changes in the fluorescence spectrum, pointing to additional dynamics as depicted in Figure 3a. On a picosecond time scale, the Soret band fluorescence vanishes and only Q band fluorescence is observed.

**Figure 4:**
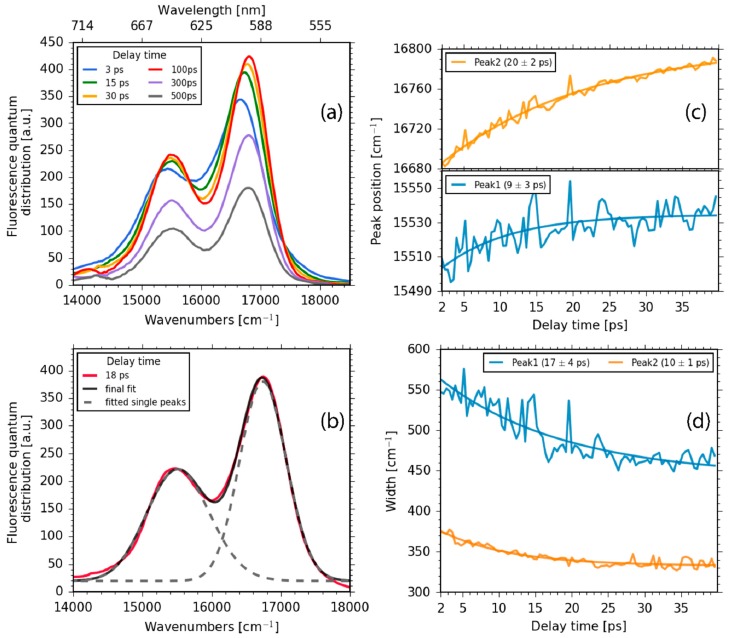
(**a**) Fluorescence spectra for selected delay times. The spectral blue-shift and narrowing of the bands is clearly visible for early delay times, while the overall decay is visible for later delay times; (**b**) Log-normal fit of the Q band fluorescence (dashed lines). lognormal(υ) = (2π)^−0.5^(συ)**^−^**^1^exp{[ln(υ) − μ]^2^/[2σ^2^]} + c, with the standard deviation σ and the mean value μ. The fit was used to determine the peak positions and peak widths (full width at half maximum (FWHM)); (**c**) Transient spectral blue-shift of the peak positions; (**d**) Narrowing of the fluorescence peaks as a function of time.

The fluorescence decay was measured up to delay times of 500 ps in the entire spectral range from 14,000 to 24,000 cm^−1^. The spectral decay of Q band fluorescence is displayed in Figure 4a and the fluorescence decay is presented in Figure 4b, simulated with a time constant of 450 ± 40 ps. The loss of fluorescence on this time scale could be explained by triplet formation, which would be consistent with previously-observed phenomena by time-resolved electron paramagnetic resonance (EPR) studies [47]. Yet, a definite assignment is not possible and the exact photophysics remain uncertain.

A closer inspection of the subtle changes of the two Q band fluorescence peaks at around 15,500 cm^−1^ and 16,700 cm^−1^ exhibits a spectral shift to higher energies. For detailed analysis, the spectrum was fitted with two log-normal distributions (see Figure 4b), revealing spectral blue-shifts with two time constants of 9 ± 3 ps and 20 ± 2 ps, respectively (see Figure 4c). This transient blue-shift is accompanied by a narrowing of the fluorescence bandwidths of the two peaks with time constants of 17 ± 4 ps and 10 ± 1 ps, respectively (see Figure 4d). The narrowing of the peaks explains the transient increase of the fluorescence in a time range from 10 to 30 ps (e.g., around 16,600 cm^−1^). We note that a transient blue-shift of the fluorescence has also been observed in other studies [48]. Several explanations have been given; the explanations that are assumed to be the most reliable are a coupling to the vibrational modes of the excited molecule or a direct interaction with solvent molecules.

### 2.2. Transient Absorption Measurements

**Figure 5:**
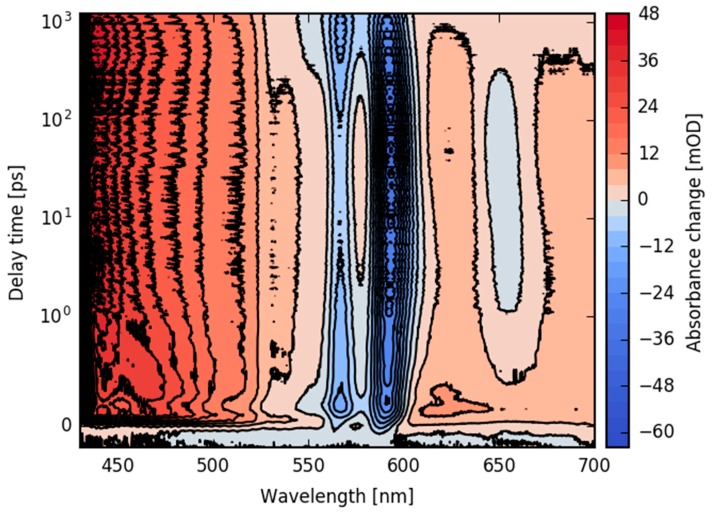
Contour plot of the absorption change upon excitation at 400 nm as a function of wavelength and of delay time from fs to 1.2 ns. The change in absorption is color-coded.

For a more detailed description of the underlying electronic dynamics, we performed transient absorption measurements using femtosecond time-resolved Vis-pump supercontinuum probe spectroscopy. Upon excitation at 400 nm, we investigated the transient dynamics from 430 to 720 nm from femtoseconds up to 1.2 ns. The absorbance changes are presented for the complete spectral range and for delay times up to 1.2 ns in a contour plot in Figure 5.

In the whole spectral range from 430 to 720 nm, we observe positive signals upon excitation up to 1.2 ns, except for the bleaching region from 560 to 580 nm and the stimulated emission region around 590 nm and at 650 nm. In the sub-picosecond time range, a change in the positive signal at wavelengths between 430 nm and 550 nm and around 630 nm is visible. These changes are followed by an increase in the stimulated emission signal at 650 nm, accompanied by the rise of stimulated emission at 596 nm. On the same time scale, the positive signal around 450 nm decays slightly and the positive signal around 540 nm rises.

Since the fluorescence of the Q band (S_1_ state) exhibits specific peaks at 596 nm and 650 nm (see Figure 1), we assign the decreasing absorption at 596 nm and 650 nm to a rise of the stimulated emission (SE) of the Q band, demonstrating Soret band to Q band transition in agreement with our time-resolved fluorescence data. As a consequence, the change in the positive signals can be assigned to the decay of the SE and the excited state absorption (ESA) of the Soret band, and rise of the ESA of the Q band. It was demonstrated that the Q band of corroles consists of two singlet excited states (S_1_ and S_2_) [44]. Our data presented here show dynamics that could be interpreted as a single Q band state. On a time scale of hundreds of picoseconds, the negative signal of the stimulated emission decays, accompanied by a decay of positive signals for long wavelengths. This results in an increase of the bleaching band. Since the stimulated emission signal decays to zero, the remaining positive and negative contributions are due to triplet absorption and bleaching signals.

**Figure 6:**
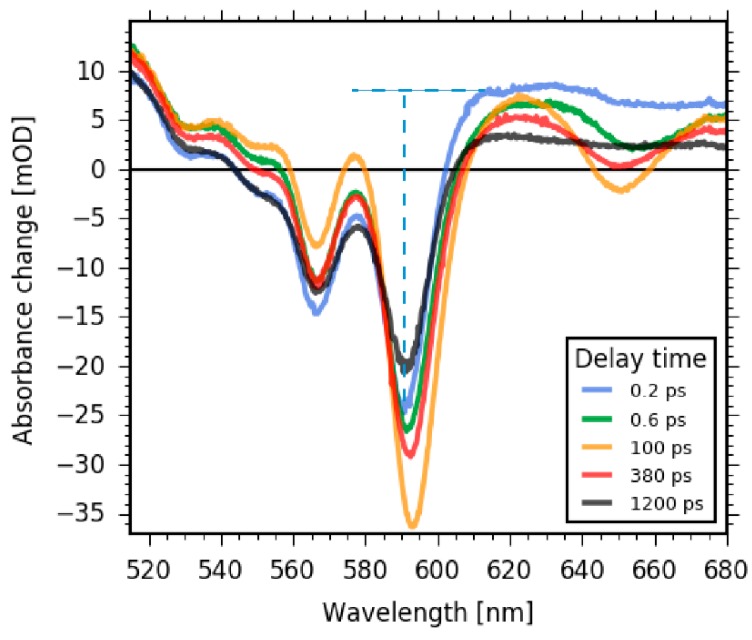
Selected absorbance difference spectra displaying the rise and decay of Q band stimulated emission (SE) signals as well as the change in excited state absorption (ESA). Over short time scales, the formation of SE at 650 nm is clearly visible. Longer delay times show the decay of SE at 650 nm and the decay of ESA around 550 nm. At early delay times (0.2 ps, blue line), the height of the Soret band ESA at 590 nm can be estimated by way of the blue dashed horizontal line and the signal strength of the initial bleaching signal at 590 nm by way of the blue dashed vertical line to be about 32 mOD.

In Figure 6, selected absorbance difference spectra are obtained for given delay times, taking cuts from Figure 5. Comparison of early delay times before Soret to Q band transition (0.2 ps, blue line in Figure 6) with delay times of Q band population (0.6 ps, and 100 ps, green and orange line in Figure 6, respectively) demonstrates the rise of the SE at around 650 nm and 596 nm, as well as the rise of ESA around 550 nm. The positive signal at around 680 nm decays with the rise of Q band ESA, indicating a stronger and broader positive band of Soret band ESA compared to Q band ESA. From the early absorbance difference spectra at 0.2 ps, when most of the population is in the Soret band and Q band SE is negligible, we can estimate the initial bleaching signal. The negative peak at 590 nm has a signal of −24 mOD, masked by the positive Soret band ESA. By assuming a broad and flat Soret band ESA at 590 nm with a signal strength of +8 mOD (dashed horizontal line in Figure 6), we can estimate the initial bleaching signal to be −32 mOD (dashed vertical line in Figure 6).

Around 100 ps after excitation, the positive signal at wavelengths between 520 nm and 685 nm due to the Q band ESA starts to decrease, accompanied by a decrease in the negative SE signal at 596 nm and 650 nm. These changes indicate a loss of SE and depopulation of the S_1_ state. Since the bleaching signals at 565 nm and 590 nm do not recover (see Figure 6) and the SE contribution at 650 nm vanishes completely, we assign this process to intersystem crossing and triplet formation. Due to the decaying of overlapping bands, the bleaching signal becomes stronger with triplet formation. Thus, we can conclude that bleaching recovery on this time scale is small or negligible. Subsequently, we assign the remaining positive signal for delay times around 1 ns to triplet state absorption.

### 2.3. Global Analysis

**Figure 7:**
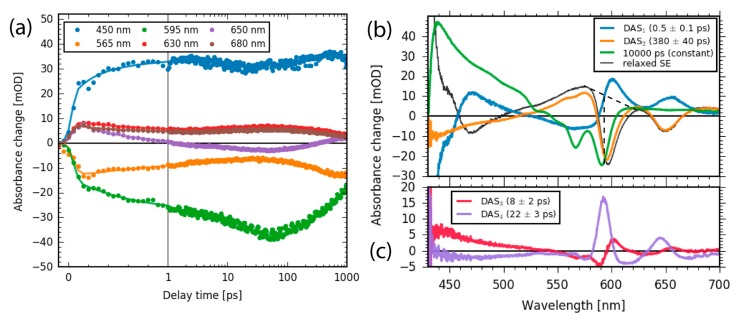
(**a**) Transient signals for selected wavelengths (dots) and fits (lines); (**b**) Decay associated spectra (DAS) describing the electronic dynamics of the Sb-corrole. DAS_1_ shows the Soret to Q band transition and rise of the SE of the Q band. DAS_2_ corresponds to triplet formation. The spectrum with a time constant of 10,000 ps represents the triplet decay and the decay of ground state bleach. The inverted sum of DAS_1_, DAS_3_, and DAS_4_ is presented as relaxed fluorescence (black line), with an added offset to match the signal of DAS_2_ at 700 nm; (**c**) two additional DAS showing cooling processes associated with a spectral blue shift and a narrowing of the respective peaks.

The complete dataset is well represented by the absorbance difference spectra and the transients at given wavelengths shown in Figure 7a. The signal to noise ratio is very good and allows for a global analysis of the complete dataset with five exponential decay functions. From the global analysis, we received five decay associated spectra (DAS) and associated decay times τ_1–5_, as displayed in Figure 7b.

The first DAS_1_ spectrum (blue line in Figure 7b) with a time constant of τ_1_ = 550 ± 50 fs can be assigned to Soret band to Q band transition. The two positive peaks at 600 nm and 655 nm imply a rise of a non-equilibrated SE of the S_1_ state. The negative contribution at around 450 nm reflects the decay of the Soret band SE and the negative contribution at around 560 nm indicates the rise of the Q band ESA, while the positive contribution at around 480 nm can be assigned to the decay of Soret band ESA. The positive signals at 600 nm in DAS_1_ (blue line in Figure 7b), DAS_4_ (violet line in Figure 7c) and the dispersive feature in DAS_3_ (red line Figure 7c) reflect the rise and relaxation of the non-equilibrated SE. The relaxed SE is presented in Figure 7b (black line) and exhibits the same signal strength in stimulated emission at 650 nm as DAS_2_. Hence, the fluorescence decay of the Q band is negligible on the time scale of our measurement and can be estimated to be smaller than 5%. This is in agreement with a comparison between the fluorescence decay time of 0.38 ns for Sb-tpfc-F_2_ with 6.6 ns for Al-tpfc-py_2_ fluorescence, resulting in a fluorescence reduction of 5%.

The signal strength of the relaxed SE can be estimated to be about 35 mOD by the negative peak at 600 nm (black dotted line in Figure 7b). We estimated the initial bleach signal to be about 32 mOD (see Figure 6), showing a similar signal strength to the relaxed fluorescence (with an error of 10%). Thus, we see no indications of a loss in population by Soret band to Q band transition. Since we do not see any contributions from the bleaching signal in the DAS_1_ spectrum, no bleaching recovery occurs on that time scale and therefore all population is transferred to the Q band.

The second DAS_2_ describes dynamics with a time constant of τ_2_ = 380 ± 40 ps. The DAS_2_ shows two negative peaks at the spectral position of the fluorescence at 595 nm and 650 nm, demonstrating the complete decay of the stimulated emission signal (also visible in Figure 5 and Figure 6). Moreover, no signatures from the bleaching signal are visible in the DAS_2_ spectrum. Thus, a new electronic state is populated that shows no stimulated emission to the ground state. Therefore, we conclude that the triplet state is formed with a time constant of τ_2_ = 380 ± 40 ps.

Moreover, a broad negative signal at wavelengths from 430 to 520 nm and a broad positive signal between 515 nm and 580 nm is visible in the DAS_2_ spectrum. The broad positive and negative contributions represent the decrease and increase of ESA from the singlet and triplet state, respectively.

Since the SE signal completely vanishes and the bleaching did not recover over longer delay times, the quantum yield for Q band to triplet state on this time scale is about 100%. This results in a total intersystem crossing (ISC) yield between 95% and 99% with a time constant of 380 ps. This is in agreement with data from Knyukshto et al. measuring ISC yields of 94% and 99% for time constants of 0.85 ns, and >0.1 ns, respectively [49].

**Figure 8:**
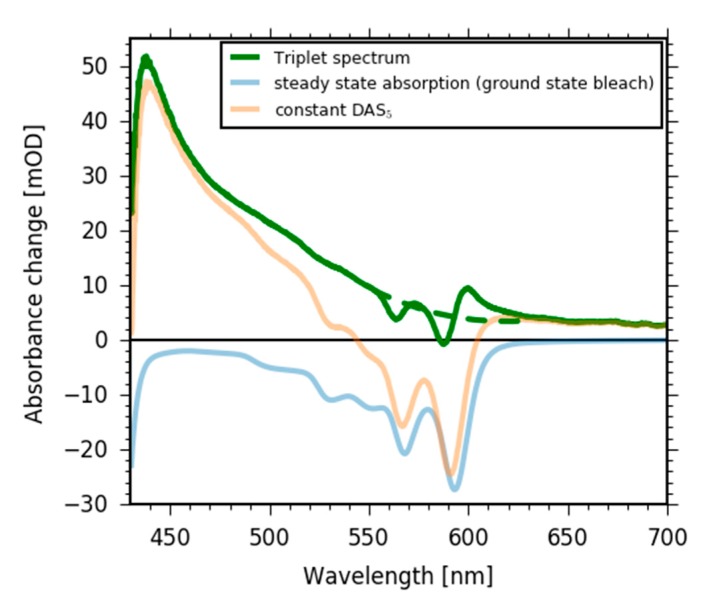
Triplet absorption spectrum (green line) calculated by subtracting the ground state bleach from the 10,000 ps DAS spectrum. The two dips and the following small positive peak are due to small calibration errors and resolution differences. The dashed line shows a possible correction of the spectrum.

Since the DAS_2_ spectrum (Figure 8, pink line) only consists of two contributions, the bleaching signal (negative) and the triplet absorption (positive), we can calculate the pure triplet absorption spectrum by adding the linear absorption spectrum (negative in Figure 8, blue line) to the DAS_2_ spectrum and minimize the spectral contributions of the complex linear absorption spectrum in triplet absorption. As visible in Figure 8 (green line), some features of the linear absorption spectrum cannot be subtracted completely from the triplet absorption spectrum; this is probably due to small deviations in wavelength calibration. Nevertheless, the shape of the triplet absorption spectrum (Figure 8, green line) shows a peak at short wavelengths and a decaying broad and flat tail down to 700 nm.

Furthermore, comparison of the DAS_1_ and DAS_2_ spectra displays a zero crossing at 520 nm for both spectra. If we assume that no triplet is generated upon Soret band to Q band transition, this indicates the same extinction coefficients at 520 nm for rising triplet absorption and decaying Q band ESA absorption (in DAS_2_), as well as the same extinction coefficients at 520 nm for rising Q band ESA absorption and decaying Soret band ESA absorption (in DAS_1_).

A closer investigation of the Q band SE for delay times between 1 ps and 50 ps exhibits a shift to higher energies (see Figure 5 and Figure 6). The two spectra, DAS_3_ and DAS_4,_ show dispersive and negative-positive-negative features at around 590 nm and 650 nm, with times constants of τ_3_ = 8.1 ± 2.0 ps and τ_4_ = 22 ± 3 ps. These features might reflect spectral shifts and a narrowing of bands at around 590 nm and 650 nm, corroborating the fluorescence data in Figure 4c,d. The DAS_4_ spectrum (Figure 7c, violet line) exhibits a prominent positive peak at 590 nm, with a small negative region at 575 nm. This can be interpreted as a blue-shift of the stimulated emission with a time constant of 22 ps. This is in perfect agreement with the fluorescence data presented in Figure 4c (orange line). Moreover, a broad positive signal at around 450 nm is visible in DAS_3_ and a broad negative signal at around 480 nm in DAS_4_. We assign these two features to spectral shifts in the Q band ESA, reflecting vibrational cooling accompanied with a narrowing and blue-shift of the Q band SE [48]. 

## 3. Discussion

Sb-tpfc-F_2_, among other corrole species, proves to have very promising photophysical properties, such as high triplet quantum yield, with possible applications in photodynamic therapy. In this study, we were able to trace electronic dynamics upon Soret band excitation of Sb-tpfc-F_2_ in real time. The use of two complementary methods lead to a consistent picture of the underlying photophysical dynamics. We were able to directly observe an internal conversion from the Soret band to Q band with a time constant of 0.5 ± 0.1 ps. On a longer time scale, intersystem crossing took place, leading to triplet generation with a time constant of 380 ± 40 ps in the transient absorption measurements and a fluorescence decay of 450 ± 40 ps in the transient fluorescence experiments. Thus, triplet formation occured on a time scale of 400 ps. The triplet yield was determined from transient absorption measurement and amounts to a about 95 to 99%. This indicates a significant spin-orbit coupling due to the antimony ion.

Relaxation processes were observed in both transient fluorescence and transient absorption measurements, displaying a blue-shift and a narrowing of the fluorescence spectra and Q band SE and ESA, respectively. These processes could be explained by the vibrational cooling of intramolecular modes via intramolecular energy relaxation or the cooling and rearrangement of solvent modes via intermolecular vibrational energy relaxation. Similar vibrational cooling time constants of about 2 ps and 20 ps were reported for heme systems upon photodissociation of carbon monoxide [50].

**Figure 9:**
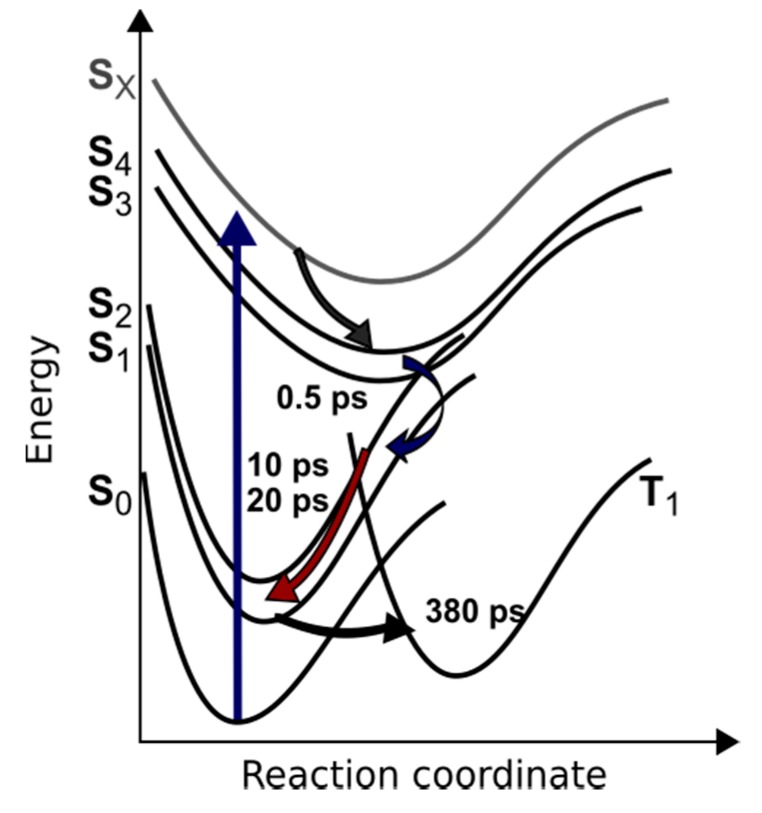
Photoreaction pathway of Sb-tpfc-F_2_ after excitation with a 400 nm pulse at the high energy side of the Soret band. Arrows indicate internal conversion from the Soret to Q band on a short time scale of 0.5 ps, with subsequent cooling processes on time scales of 10 ps and 20 ps. Intersystem crossing from the S_1_ state to the T_1_ state was observed with a time constant of 400 ps.

The resulting decay paths are depicted in Figure 9. However, further investigations on different corrole complexes are needed to fully understand the effect of different metal ions and ligands. This knowledge is crucial to further fine-tune the photophysics of corrole complexes in the future.

## 4. Materials and Methods

The transient absorption experiment was performed using a Ti:sapphire Laser Legend USP from Coherent (Santa Clara, CA, USA), with a center wavelength of 800 nm and a pulse duration of 80 fs. The repetition rate is 1088 Hz and the energy of the pulse is approx 2.6 mJ. The setup consists of two parts, for pump and probe pulse generation. The pump pulse is generated by second harmonic generation in a BBO (β-bariumborate) crystal, giving a pump pulse energy of 6 µJ attenuated to about 0.5 μJ. The change in absorption between the pumped and unpumped sample was measured by using a mechanical chopper blocking every second pump pulse. A parallel (A_pa_) and perpendicular (A_pe_) signal was obtained by rotation of the pump pulse polarisation using a rotatable λ/2 plate. Isotropic absorbance changes (A_iso_) were calculated by A_iso_ = (A_pa_ + 2 A_pe_)/3.

The broad supercontinuum probe pulse was generated by focusing the fundamental in a 1 cm fused silica cuvette filled with water. The resulting supercontinuum pulse covered the whole visible spectrum from 390 up to 750 nm. Due to saturation, we used an HR 800 mirror (Laseroptik, Garbsen, Germany) and an optical SCHOTT BG 26 filter (Schott AG, Mainz, Germany) to cut off wavelengths longer than 750 nm. The probe pulse was delayed using a translation stage M535.22 from Physik Instrumente (Karlsruhe, Germany) with an accuracy of 0.1 μm.

After passing the sample, the probe beam was coupled in an optical spectrometer (Shamrock from Andor, Entwicklungsbüro Stresing, Berlin, Germany). The system response was better than 100 fs (typically ~80 fs) [51]. The generated probe pulse supercontinuum exhibited a strong chirp, resulting in a shift of time-zero as a function of the wavelength. The data were time-zero corrected by modeling the chirp with a third order polynomial [51].

Femtosecond fluorescence spectra were measured with a broadband up-conversion setup FLUPS (fluorescence up-conversion spectroscopy, LIOP-TEC, Radevormwald, Germany) [52,53,54]. The sample was excited with 400 nm pump pulses. Fluorescence was collected with an off-axis Schwarzschild mirror objective and re-focused onto a BBO crystal (100 μm thick, EKSMA Optics, Vilnius, Lithuania), where it was up-converted (type II) with 1340 nm gate pulses. A time resolution of 100 fs, as measured at full width at half maximum (FWHM) of solvent Raman signal, was achieved, despite the large interaction angle (21°), by tilting the gate pulse front [52]. For the measurements in this work, the crystal was set to case A conditions as described in Gerecke et al. [54]. Up-converted fluorescence was detected, simultaneously at all relevant wavelengths, by a spectrograph equipped with a CCD camera (Andor DU420 BU, Andor Technology Ltd., Belfast, UK). Transient spectra were collected in 20 fs, 400 fs and 10 ps steps up to 500 ps, integration over 0.5 s was used at each step of a time scan and the results from four scans were averaged. Finally, corrections for the dispersion of group velocity and photometric response were applied. 

Sb-tpfc-F_2_ was prepared according to Luobeznova et al. [22]. The sample was dissolved in toluene.
